# Metabolomic Phenotyping of Gliomas: What Can We Get with Simplified Protocol for Intact Tissue Analysis?

**DOI:** 10.3390/cancers14020312

**Published:** 2022-01-09

**Authors:** Paulina Zofia Goryńska, Kamila Chmara, Bogumiła Kupcewicz, Krzysztof Goryński, Karol Jaroch, Dariusz Paczkowski, Jacek Furtak, Marek Harat, Barbara Bojko

**Affiliations:** 1 Department of Pharmacodynamics and Molecular Pharmacology, Faculty of Pharmacy, Collegium Medicum in Bydgoszcz, Nicolaus Copernicus University in Toru, 85-089 Bydgoszcz, Poland; gorynska@cm.umk.pl (P.Z.G.); kamila_chmara@wp.pl (K.C.); gorynski@cm.umk.pl (K.G.); karol.jaroch@cm.umk.pl (K.J.); 2 Department of Inorganic and Analytical Chemistry, Faculty of Pharmacy, Collegium Medicum in Bydgoszcz, Nicolaus Copernicus University in Torun, 85-089 Bydgoszcz, Poland; kupcewicz@cm.umk.pl; 3 Neurosurgery Unit, 10th Military Research Hospital and Polyclinic, Independent Public Healthcare Centre, 85-681 Bydgoszcz, Poland; darek_paczkowski@vp.pl (D.P.); jacek.furtak2019@gmail.com (J.F.); 4 Department of Neurosurgery and Neurology, Faculty of Health Sciences, Collegium Medicum in Bydgoszcz, Nicolaus Copernicus University in Torun, 85-168 Bydgoszcz, Poland

**Keywords:** glioma, SPME, brain tumor, IDH, 1p19q codeletion, metabolomics

## Abstract

**Simple Summary:**

The diagnostic protocol for gliomas is based on histological examination and the determination of genetic biomarkers. However, examining molecular biomarkers in cancer tissue is usually labor-intensive and time-consuming when a homogenization step is involved. Therefore, this diagnostic approach has not been fully explored to date. The present study seeks to validate the applicability of solid-phase microextraction (SPME), or chemical biopsy, as a new approach for fast and simple sampling and sample-preparation in the surgery room prior to the application of metabolomic analysis to identify biomarkers. To this end, the metabolomic profiles of brain tumors were compared with genetic biomarkers and the results of histological analysis in order to identify changes of molecular metabolites of statistical significance. The findings of this study indicate that the proposed approach provides complementary information to current diagnostic methods and has the potential to be a valuable on-site analytical tool in future applications.

**Abstract:**

Glioblastoma multiforme is one of the most malignant neoplasms among humans in their third and fourth decades of life, which is evidenced by short patient survival times and rapid tumor-cell proliferation after radiation and chemotherapy. At present, the diagnosis of gliomas and decisions related to therapeutic strategies are based on genetic testing and histological analysis of the tumor, with molecular biomarkers still being sought to complement the diagnostic panel. This work aims to enable the metabolomic characterization of cancer tissue and the discovery of potential biomarkers via high-resolution mass spectrometry coupled to liquid chromatography and a solvent-free sampling protocol that uses a microprobe to extract metabolites directly from intact tumors. The metabolomic analyses were performed independently from genetic and histological testing and at a later time. Despite the small cohort analyzed in this study, the results indicated that the proposed method is able to identify metabolites associated with different malignancy grades of glioma, as well as IDH and 1p19q codeletion mutations. A comparison of the constellation of identified metabolites and the results of standard tests indicated the validity of using the characterization of one comprehensive tumor phenotype as a reflection of all diagnostically meaningful information. Due to its simplicity, the proposed analytical approach was verified as being compatible with a surgical environment and applicable for large-scale studies.

## 1. Introduction

Gliomas account for a significant portion of primary brain tumors, and they possess an analogous histological structure to normal glial cells (i.e., ependymal, astrocytes, and oligodendrocytes). There is a wide spectrum of malignancy in each group of gliomas. Among the astrocytic group, glioblastoma multiforme is the most common malignant brain tumor in humans and is characterized by short survival times due to its metabolic activity and poor response to therapy. The transformation of a tumor from benign to malignant is accelerated by genetic mutations that stimulate uncontrollable cell proliferation, transform cell death programs, and alter cellular metabolism [1,2]. One of the most crucial aspects of identifying an innovative therapeutic target or potential biomarkers is the discovery of a tumor’s metabolic pathways [3,4]. Based on numerous studies of glioma cell lines, it was proven that low levels of glucose oxidation in the mitochondrial citric acid cycle (CAC) contribute to high rates of glycolysis and glutaminolysis in glioblastoma multiformes [5]. Currently, a typical medical intervention for malignant brain tumors will consist of tumor resection (to the greatest degree possible) followed by temozolomide therapy with radiation [6]. The first step in this process is critical, as the degree of the excision is the most significant factor in determining the patient’s survival rate. Indeed, findings have shown that patients who underwent total resection responded better to temozolomide therapy compared to those who only received a partial resection [7]. Despite the range of therapeutic procedures that can be deployed against malignant gliomas, their prognosis remains unsatisfactory. The WHO guidelines for brain tumor classification published in 2016 affirm the indispensable role of genetic profiling in diagnosing and medicating gliomas. The latest update from 2021 is related to the analysis of many molecular profiles [8]. In particular, the statuses of isocitrate dehydrogenase 1 mutation (IDH1) and 1p19q codeletion are two crucial indicators in determining the genetic profiles of gliomas [9]. The IDH1 mutant is associated with better prognoses for patient survival than the IDH1 wild-type that is common to anaplastic astrocytoma and glioblastoma. As such, the determination of the tumor’s genetic profile provides more predictive power compared to histological diagnoses of high-grade astrocytomas [10,11]. However, in order to define a tumor’s phenotype according to the latest WHO recommendation, it is necessary to perform a series of time-consuming tests. One of the most common classification methods used to analyze tumors after surgery or biopsy consists of microscopic examination (i.e., histopathology) followed by analysis of isocitrate dehydrogenase 1 mutation status (IDH1) and the presence of 1p19q codeletion. However, this technique is not able to provide comprehensive insight into the tumor’s entire biochemistry, which would enable molecular-level processes to be distinguished from one another.

The ability to detect small molecules characteristic for gliomas during surgery or biopsy would allow cancer phenotypes to be determined more quickly in individual patients, which would effectively reduce analytical time and allow therapy to commence immediately after tumor resection. The study of small molecules, known as metabolomics, provides valuable information about the system under study, as it reflects all changes occurring at the genome, transcriptome, and proteome level, as well as the environmental factors that influence the system at these levels. Metabolomics is frequently employed to identify potential biomarkers or reveal the underlying mechanisms of a disease’s pathology using biofluids, cells, or tissue as a sample matrix. Therefore, metabolomics can be a valuable tool for personalized medicine, as well as in screening for risk factors among specific populations [12].

Sample preparation is the most crucial step in metabolomics analysis, especially when working with biological samples. Tissue processing is more complex, and therefore time consuming and labor intensive, than working with biofluids. Under traditional sample-preparation protocols, sample collection is immediately followed by metabolism quenching, weighting, homogenization, (multi-)solvent extraction, evaporation, and reconstitution. Given this extensive process, traditional sample-preparation methods are simply unsuitable for on-site sampling. Nowadays, there are numerous alternative diagnostic methods providing the opportunity to minimize the time required for the results of histopathological/genetic examination and assessing the boundary between a healthy and tumor tissue during surgery. Many research groups are working on solutions in this area. So called “intelligent knife” or “iKnife” developed by Prof. Takats’ group is a modification of surgical scalpel routinely used in clinical practice. It enables surgeon to monitor in real time whether the cut tissue is characterized as malignant, healthy or borderline based on the analysis of the smoke that’s produced when heat cuts tissue. The technique was already tested for the analysis of breast, ovarian, and cervical cancer [13,14,15,16]. Another technique widely tested for its suitability to the intra-surgical assessment of given tissue is desorption electrospray ionisation (DESI) coupled to mass spectrometry. It allows for molecular characterization of the tissue and recognition of tumor margin [17,18]. DESI is not applicable for in vivo use, so analysis is preceded by the removal of a piece of tissue with standard surgical tools, e.g., combination of surgical forceps and CUSA (cavitron ultrasonic surgical aspirator). The approach offers even three-dimensional images, but the overall time of the analysis (from sampling to final image) cannot be considered as rapid testing. However, developments in DESI systems clearly show a trend towards faster analysis [19]. Recently, a DESI-MS system as well as extraction nanoelectrospray ionization were successfully tested for intraoperative identification of IDH mutation status by measuring ion intensities of 2-hydroxyglutarate (2-HG) from tumor cores [20,21,22,23]. There are also laser-based techniques, such as Spider Mass or PIRL-MS, tested for their potential in the diagnosis of ovarian, skin cancer, sarcoma, and medulloblastoma among others [24,25,26]. One of the least invasive intraoperative methods is MasSpec Pen, which utilizes a drop of water for fast 1 s extraction (mixing with tissue fluid followed by its aspiration to mass spectrometer). So far, the MasSpec Pen has been used to analyze breast, thyroid, ovary, lung, and carcinoma cancer [27]. In the current work, we propose the use of solid-phase microextraction (SPME), or chemical biopsy, for the on-site sampling of human brain tumors in order to enable metabolomic phenotyping and the identification of discriminating metabolites among these tumors. The main advantages of SPME are: (1) its simple sampling protocol, which does not require tissue weighting or the use of solvents for analyte extraction, and (2) its lack of physical tissue consumption, which enables all types of biological material to be used for routine analysis (e.g., histological or genetic testing). The SPME device used in this research consists of a microprobe coated with a specially designed biocompatible extraction phase that absorbs the small molecules but prevents the adhesion of macromolecules or cells. The on-site portion of the protocol consists of inserting the SPME probe into the resected tumor for a set time period, followed by rinsing with water for a few seconds, and finally storing it in a vial for transportation. The current work is a proof-of-concept study that demonstrates the proposed strategy’s ability to identify a representative range of metabolites characterizing brain tumor phenotypes. As such, it possesses tremendous potential for use in large-scale screening studies and rapid on-site personalized analysis.

## 2. Materials and Methods

### 2.1. Chemicals and Materials

All solvents used for analysis (i.e., acetonitrile, methanol, water, formic acid, and ammonium acetate) were LC-MS grade and purchased from Sigma Aldrich (Poznań, Poland). The chromatographic experiments were performed using a Discovery HS F5 (100 x 2.1 mm, 3 µm) column, which was an in-kind gift from MilliporeSigma/Supelco (Bellefonte, PA, USA), and an HILIC column (Luna HILIC 100 mm × 2.0 mm, 3 μm), which was purchased from Phenomenex (Shimpol, Poland).

### 2.2. Patients

All experiments were approved by the Bioethics Committees at the Collegium Medicum in Bydgoszcz, Nicolaus Copernicus University in Torun (KB 628/2015), and informed consent was obtained from all patients prior to participation. The patients underwent tumor resection at the 10th Military Research Hospital in Bydgoszcz, Poland, between December 2016 and February 2017. In total, 38 brain tumor samples were collected (Table 1). The inclusion criteria were as follows: age > 18, patients with glioma or meningioma, good quality of life (Karnofsky Performace Status Scale > 70). Exclusion criteria: age < 18, inability or unwillingness to participate in the study or to sign the informed consent form, tumor size less than 1 cm in diameter, high internal burden increasing the risk of surgery. Each sample underwent histological analysis, where it was categorized as either astrocytoma, glioblastoma, ependymoma, or oligodendroglioma, followed by additional genetic testing (Table 1).

### 2.3. Sample Preparation

**Figure 1:**
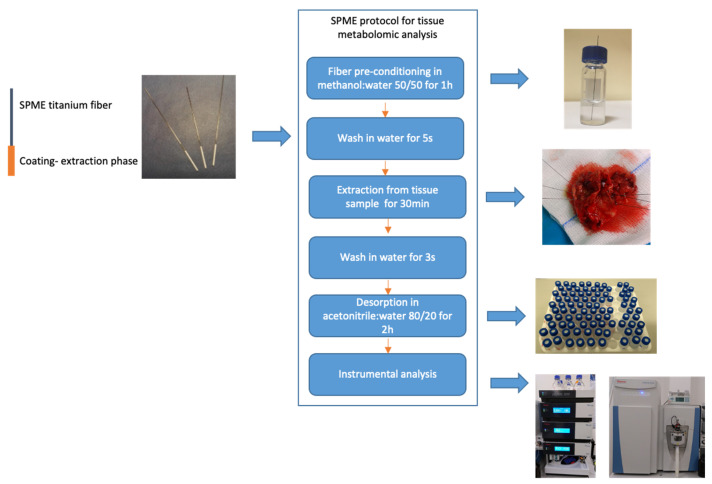
Sample-preparation protocol using solid-phase microextraction in brain tumors analysis.

The metabolomic analysis were performed independently from genetic and histological testing and at later time. All procedures and experimental conditions were adopted from Gorynska et al. [28]. The SPME probe was 4 cm long and consisted of a nickel-titanium alloy support coated with 7 mm of mixed-mode extraction phase (i.e., C18 and benzoic acid). As illustrated in Figure 1, the SPME protocol comprised four main steps: coating pre-conditioning, extraction, rinsing and desorption. The fiber coating was preconditioned statically in a 1.5 mL methanol:water (1:1, *v*:*v*) mixture for 1 h, while the extraction step was performed at the hospital directly following the tumor resection. Just before sampling, the fiber was immersed in water for 5 s to remove any remaining methanol, which could lead to protein precipitation on the coating. Sampling was performed by inserting the probe into the brain tumor for 30 min immediately following resection. After sampling had been completed, the fiber was extracted and rinsed in 1.5 mL of water for 3 s with no agitation, then immediately placed in a closed vial and transported back to the laboratory in a Styrofoam box filled with frozen ice packs. Once back at the laboratory, the vials containing the probes were stored in a freezer at −30 °C until analysis. On the day of analysis, the probes were removed from the freezer and desorbed for 120 min at 25 °C in 0.3 mL of acetonitrile:water (4:1, *v*:*v*) with agitation at 1200 rpm on a BenchMixer™ MultiTube Vortexer (Benchmark Scientific, Edison, Sayreville, NJ, USA). To eliminate signals from compounds other than those derived from the tumor, blank extracts (i.e., controls) of the solvents and environment were used, with the detected signals being excluded from analysis in the data processing step.

### 2.4. LC-MS/MS Analysis

Instrumental analysis was performed using an ultra-high-performance liquid chromatography system (UltiMate 3000, Thermo Fisher Scientific, Bremen, Germany) coupled to a high-resolution mass spectrometer (Q Exactive Focus Orbitrap, Thermo Fisher Scientific, Bremen, Germany). The analytes were separated using a reversed-phase pentafluorophenyl (PFP) column (Discovery HS F5 100 × 2.1 mm, 3μm, Supelco, Bellefonte, PA, USA) and a hydrophilic interaction chromatography column (Luna HILIC 100 mm × 2.0 mm, 3 μm, Phenomenex, Torrance, CA, USA). The gradients for both methods were adopted from [29]. All chromatographic conditions and MS parameters have been detailed in a previous work [30].

To control the instrument’s performance during the analysis, quality control (QC) samples were run every 8–10 injections. The QC samples were prepared by mixing 10μL aliquots of each sample included in the analysis and injecting the samples randomly. The instrument was calibrated using external calibration (Pierce™ LTQ ESI Positive Ion Calibration Solution and Pierce™ Negative Ion Calibration Solution, Thermo Scientific, San Jose, CA, USA), with calibration being performed every 48 h. This resulted in a mass accuracy of <2 ppm.

### 2.5. Data Processing and Statistical Analysis

Data processing was conducted using the Compound Discoverer 2.1 software package with the following filtering parameters: a lower RT limit of 1 for analysis using the PFP and HILIC columns; upper RT limits of 34 and 18 for analysis with the PFP and HILIC columns, respectively; a minimum QC coverage of 50%; a maximum QC area RSD of 30%; a minimum peak intensity of 1,000,000; a minimum precursor mass of 100 Da; a maximum precursor mass of 5000 Da; an S/N of 3; a minimum collision energy of 0; a maximum collision energy of 1000; an S/N Threshold (gap filling) of 1.5; an RT tolerance of 0.1 min; a maximum RT shift of 2 min; a mass tolerance of 5 ppm; and an intensity tolerance of 30%. The detected discriminant metabolites were identified based on fragmentation-pattern matching using mzCloud software.

Two approaches were applied to obtain compounds or a panel of compounds responsible for discriminating between groups of patients with specific histological or genetic types of tumor. The first approach employed L1-penalization for variable selection and the orthogonal partial least squares discriminant analysis (OPLS-DA) algorithm. All calculations were conducted using PLS Toolbox (Eigenvector Research Inc., Manson, WA, USA) and MatLab 2020b software (MathWorks, Natick, MA, USA). LASSO was applied to identify the relevant in-dependent variables that significantly affect the classification/discrimination. The variable coefficients (loadings) of the PLS components represent a measure of how much a variable contributes to the discrimination of the different sample groups. Model dimensionality, defined as the number of PLS factors (latent variables-LV), was estimated as a compromise between cross-validation error, number of misclassifications (NMC), and area under the receiver operating characteristic (AUROC).

The second method entailed the use of ANOVA and Tukey’s post hoc test, *p*-value adjustment via the Benjamini–Hochberg method to minimize the false-discovery rate, and group comparisons using ratio and fold-change values to select compounds demonstrating major statistical significance in relation to the divisions between analyzed groups. Descriptive statistics accounted for the minimum, maximum, median, first quartile (Q1), third quartile (Q3), mean, and standard deviation (SD) for the peak areas. As described above, pre-processing and data acquisition were performed using Compound Discoverer 2.1 software, while Matlab was used for statistical data analysis.

## 3. Results

### 3.1. Sampling Procedure and Analysis

**Figure 2:**
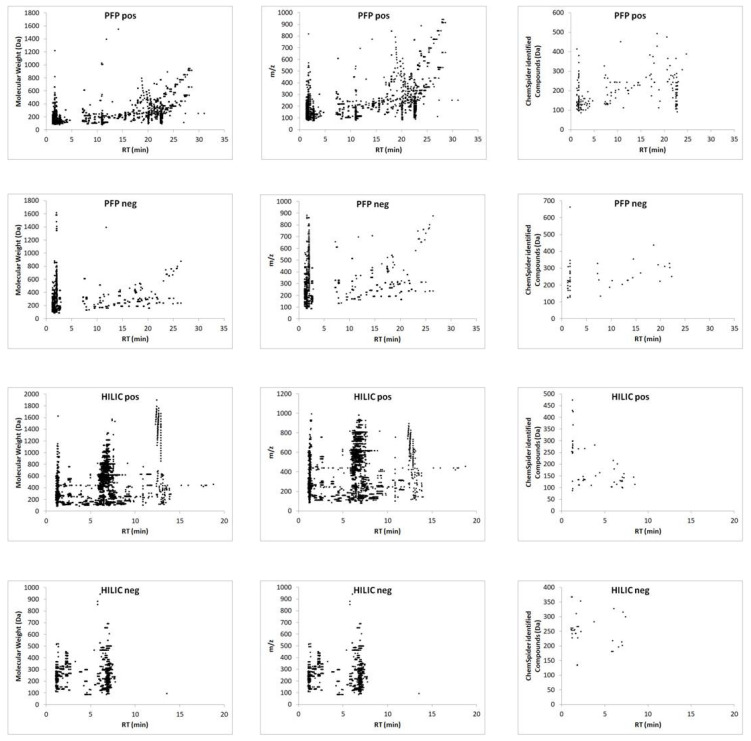
Ion map of extracted metabolites defined by molecular weight and m/z, and Chemspider-identified compounds from the PFP and HILIC column in positive and negative ionization mode followed by LC–MS analysis.

The proposed method was employed to sample all resected tumors, with no difficulties being observed in relation to tissue penetration with the microprobe. After desorption, the extracts were subjected to instrumental analysis. A comparison of the data obtained from all combinations of chromatographic separations and ionization modes revealed that the use of the reversed-phase column and positive ionization enabled the detection of the highest number of small molecules (Figure 2, Tables S1 and S2), and thus the most efficient differentiation between analyzed groups.

### 3.2. Metabolomic Changes in Tissue Samples of Gliomas Compared to Meningiomas

**Figure 3:**
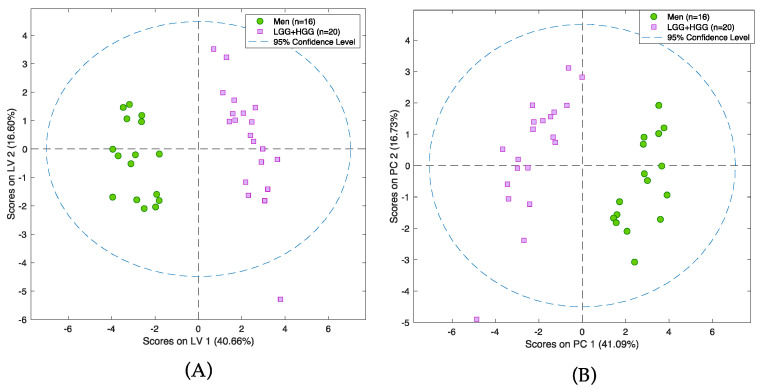
PCA scores plot (**A**) and PLS-DA model (**B**) presenting differences between group of glioma and group of meningioma samples. Pink squares represent patients with meningioma and green circles patients with glioma. Analysis was performed on a PFP column in positive ionization mode.

The first step of the analysis aimed to determine whether the proposed strategy is able to distinguish between two tumors of completely different histological origin, as well as their degree of malignancy. For this purpose, gliomas and meningiomas (MEN) were chosen. In addition, the glioma group consisted of high- and low-grade-malignancy (HGG and LGG, respectively) tumors. As noted earlier, gliomas are highly malignant tumors that arise from glia cells; in contrast, meningiomas are benign tumors that form from meninges. Therefore, the separation between these groups needs to be significant, despite intra-group variability. This study employed two statistical methods of variable selection: one consisting of filter techniques that act on the intrinsic properties of the data itself, while ignoring the subsequently used classification or prediction algorithm (ANOVA); and another consisting of embedded techniques, wherein variable selection is built into the prediction algorithm (LASSO) [31]. Both approaches yielded satisfactory results, with the LASSO method successfully identifying a set of compounds that drive the separation between the selected groups of tumors (Figure 3A,B). Validation details relating to the models are presented in Tables S3 and S4. Among others, statistically significant changes of 17 molecules and several endogenous compounds previously reported in the literature (i.e., lysine, aspartic acid, creatine, and citrulline) were identified (Table S5).

ANOVA tests were able to identify 43 metabolites, several of which having been previously described in the literature (e.g., cystathionine, aspartic acid, arginine, and lysine); these results indicated that the ANOVA-based approach had high statistical significance in differentiation meningiomas (MEN) and gliomas (LGG and HGG) (Table S6). Whereas patients with glioma tumors exhibited significantly increased levels of cystathionine (*p*-value = 0.0065), patients with meningiomas had higher levels of other amino acids, such as aspartic acid, lysine, and arginine. The tracking values of statistical significance included: *p*-values calculated based on T-test; corrected *p*-values considering the false discovery rate as determined by Benjamini–Hochberg test; and the ratio of the area of a given peak in the glioma sample group to the corresponding peak area in the meningioma group. The identities of the compounds detected in both analyses were confirmed based on the comparison of the fragmentation patterns in the experimental spectra and MzCloud database.

### 3.3. Metabolomic Differences of Glioma Samples of Various Histological Types

**Figure 4:**
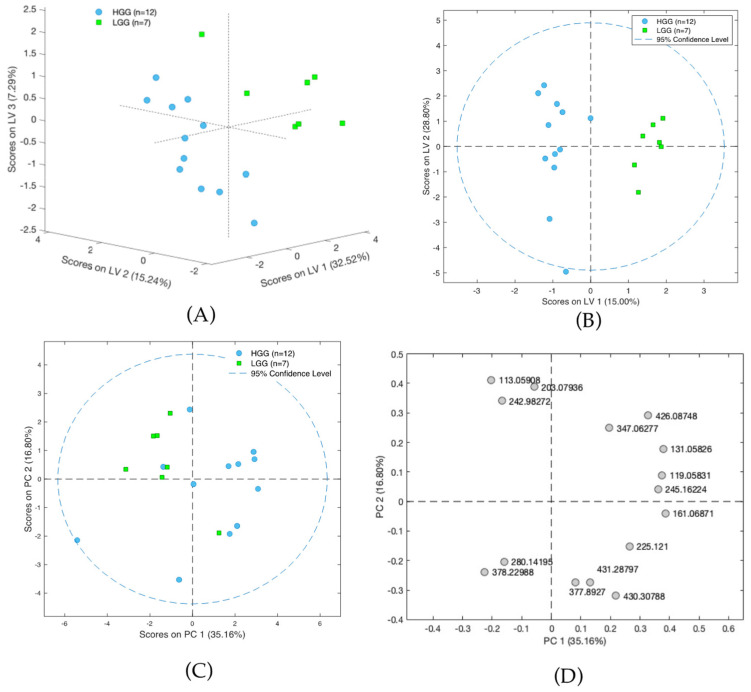
(**A**) Scores plot of the PLS-DA model showing differences between groups of high-grade-malignancy gliomas (HGG) and low-grade-malignancy gliomas (LGG). Results presented on three first latent variables. (**B**) Scores plot of the OPLS-DA two-class model of LC-MS data. The labels correspond to patients with high-grade-malignancy gliomas (blue circles) and those with low-grade-malignancy gliomas (green squares). (**C**) PCA scores plot showing data for patients with high-grade-malignancy gliomas (blue squares) and those low-grade-malignancy gliomas (green circles). (**D**) PCA loadings plot for HGG and LGG patients.

In the next step, the analysis examined only on the glioma samples, with satisfactory results being obtained for the differentiation of the LGG and HGG groups (Figure 4A–D). The results obtained with LASSO indicated that separation was driven by statistically significant changes of 16 molecules, with L-2-aminoadipic acid exhibiting the highest HGG/LGG ratio (>15) (Table S7). Other confirmed metabolites present at lower levels in low malignancy gliomas included aminolevulinic acid and threonine, while creatinine was present in higher levels in LGG compared to HGG. Tentatively identified lipid metabolites indicated the potential involvement of vitamin D3 derivatives and carnitines in cancer malignancy processes. However, it must be emphasized that none of the aforementioned compounds were found to be statistically significant following the application of FDR correction. This could be a result of the small size of the groups included in the studies and high inter-group variability and it will be verified in future extended investigations.

**Figure 5:**
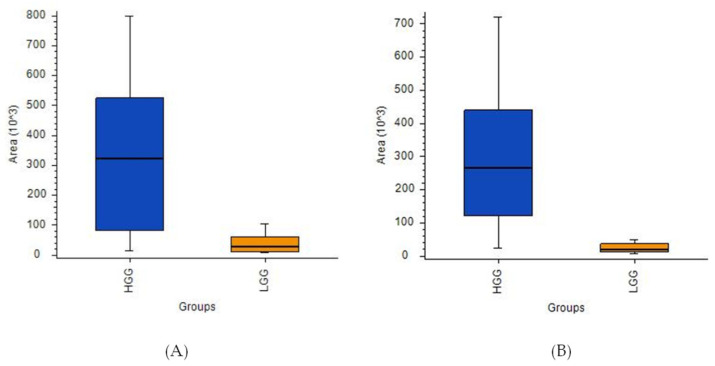
Box-and-whiskers plot for (**A**) L-2-aminoadipic acid and (**B**) propionylcarnitine.

A comparative analysis of HGG and LGG patients using ANOVA indicated the presence of 13 compounds with high statistical significance (Figure 5A,B, Table S8). Ultimately, propionylcarnitine proved to be the best indicator for comparing these two tumor groups based on the [HGG]/[LGG] ratio (14.36) and a *p*-value of < 0.001. However, the corrected *p*-value did not meet the significance criteria for this or any of the other metabolites initially identified as discriminant features, namely phenylalanine, proline, tyrosine, uric acid, and 2-aminoadipic acid (the latter was also selected by LASSO).

### 3.4. Metabolomic Changes in Tissue Samples with or without Isocitrate Dehydrogenase 1 and 2 Mutation

A group of 19 glioma samples was used to conduct a comparative study of mutated and wild-type IDH. Genetic testing was performed in accordance with the 2016 World Health Organization Classification of Tumors of the Central Nervous System. The characteristics of the tumors collected for this analysis are summarized in Table 1.

**Figure 6:**
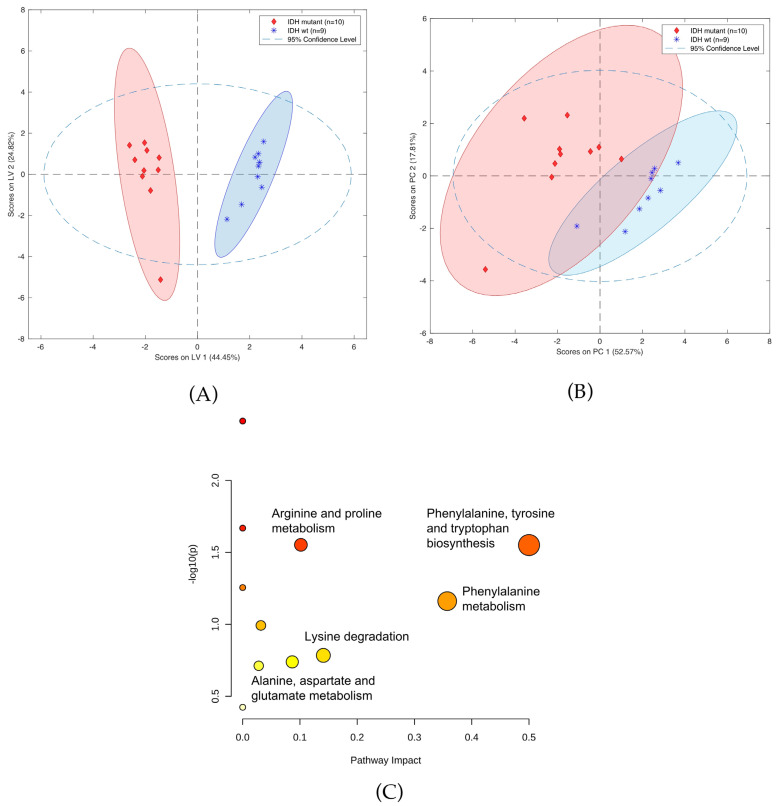
(**A**) OPLS-DA two-class model (**B**) and PCA plot of LC-MS data from patients with (blue circles) and without IDH mutation (green squares). (**C**) Pathway analysis of metabolites present in patients due to IDH mutation.

A panel of 12 compounds differentiating cancer samples with confirmed or denied IDH mutation was selected using the LASSO method. This panel of compounds included creatinine, threonine, carnitine, and neurine (Table S9). Notably, it was not possible to identify six of the detected compounds, as they were not present in any of the data bases. Figure 6 shows OPLS-DA and PCA plots representing the separation of the investigated groups, while a comparison of the masses selected by LASSO with the use of HILIC and PFP columns is presented in Table S10. Furthermore, the validation parameters of models describing patients with codeletion 1p19q are presented in Table S11 and Figure S1. Pathway analysis based on statistically significant changes of metabolic features was also performed, with results indicating that the most important metabolic pathways for the presence of the IDH mutation included phenylalanine, tyrosine, and tryptophan biosynthesis; phenylalanine biosynthesis; arginine and proline metabolism; lysine degradation; and alanine, aspartate, and glutamate metabolism (Figure 6C).

**Figure 7:**
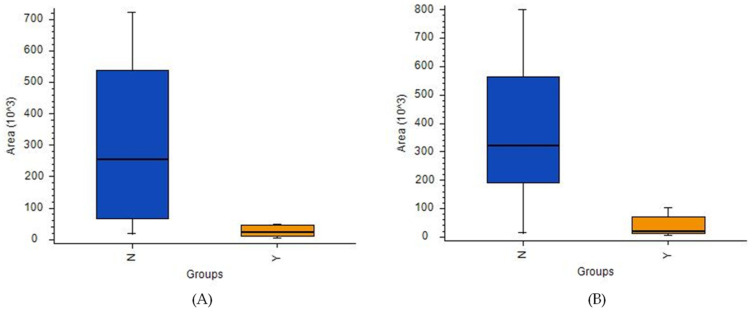
Box-and-whiskers plot for (**A**) propionylcarnitine and (**B**) 2-aminoadipic acid. (Abbreviations: N–IDH wild-type; Y–IDH mutant).

While an ANOVA detected many metabolites with satisfactory [IDH]/[no-IDH] ratios and *p*-values < 0.01 or 0.05 (Table S12), none of these metabolites’ *p*-values were statistically significant after correction. Notably, two compounds were present in IDH mutants at levels > 10 times higher than in the IDH wild-type: 2-aminoadipic acid (ratio: 17) and propionylcarnitine (ratio: 11) (Figure 7A,B, respectively).

### 3.5. Metabolomic Changes in Tissue Samples with and without 1p19q Codeletion

**Figure 8:**
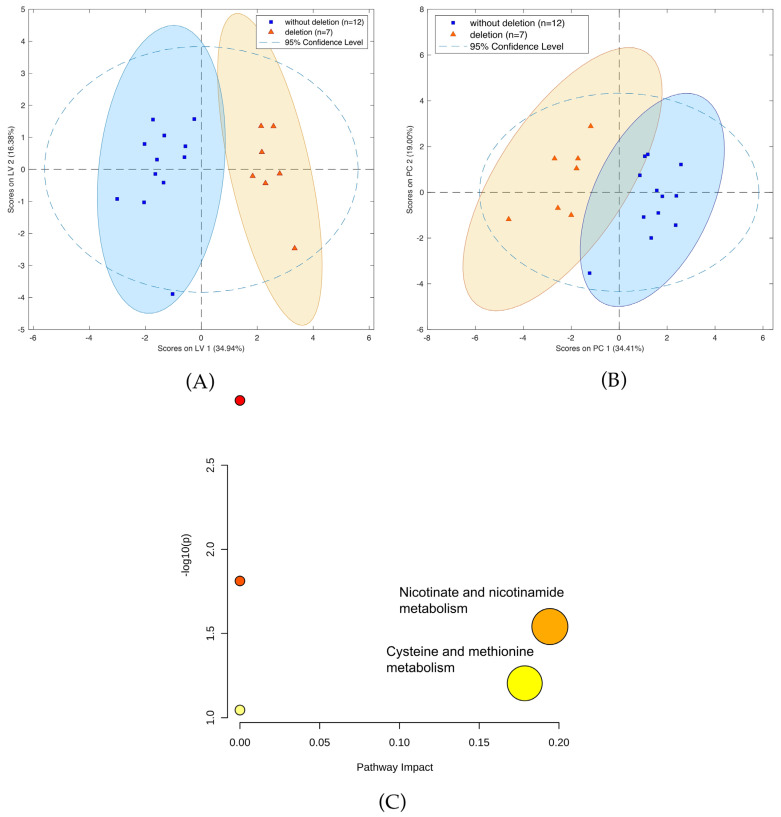
(**A**) OPLS-DA two-class model and (**B**) PCA plot of LC-MS data from patients with (blue circles) and without 1p19q codeletion (green squares). (**C**) Pathway analysis of metabolites present in patients due to1p19q codeletion.

A comparison of patients with and without 1p19q codeletion was also performed. Calculations using LASSO resulted in the identification of 13 compounds (Table S13) separating the two cohorts (Figure 8A,B). In particular, two metabolic pathways were found to have the greatest impact in determining the presence of 1p19q codeletion: nicotinate and nicotinamide metabolism; cysteine and methionine metabolism (Figure 8C). Furthermore, six compounds, namely threonine, neurine, nicotinamide, oxidized glutathione, and the vitamin D3 derivatives 7alpha-Hydroxy-3-oxo-4-cholestenoate and monoglyceride, showed greater downregulation in mutants compared to the wild-type. Conversely, up-regulation was observed for sn-glycero-3-Phosphoethanolamine and glycerylphosphorylethanolamine, as well as for one unidentified compound with a molecular weight of 163.1207. A comparison of masses of the compounds selected by LASSO using HILIC and PFP columns is presented in Table S14, while the validation parameters of the models describing patients with codeletion 1p19q are presented in Table S15 and Figure S2.

**Figure 9:**
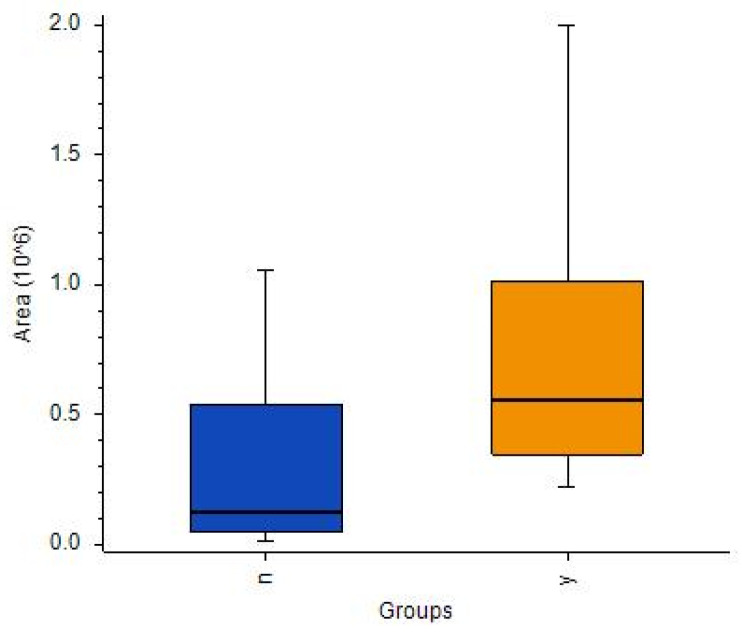
Box-and-whiskers plot for cystathionine in samples with detected 1p19q codeletion (orange) and 1p19q wild-type (dark blue). (Abbreviations: N–1p19q wild-type; Y–1p19q codeleted).

An ANOVA indicated eight compounds (Table S16), but only one (cystathionine) was identifiable based on its fragmentation spectra with *p*-value < 0.05 and a [no codeletion/codeletion] ratio of 0.223. The corrected *p*-value was 0.16 (Figure 9).

## 4. Discussion

Routine procedures performed on resected brain tumors rarely include extensive biochemical tissue analysis. However, in recent years, many molecular and genetic biomarkers have been identified as noninvasive factors in the diagnosis of gliomas, including glutamate [32], ATIA [33], cathepsin D [34], GADD45 [35], YKL40 [36], Ki67 [37], MIP-1 [38], and CDKN2A [39]. In particular, isocitrate dehydrogenase 1 and 2 (IDH1 and IDH2) mutation [40,41] and 1p/19q codeletion [42] have been recognized as promising predictive molecular markers, resulting in their inclusion in the 2016 World Health Organization (WHO) classification of gliomas [9]. However, none of these biomarkers enable a comprehensive phenotype assessment that accounts for the tumor’s histological properties, as well as associated genetic mutations.

Although previous metabolomics/lipidomics studies have sought to discover biomarkers in brain cancers, particularly gliomas, these studies primarily focused on phenotyping particular mutants (e.g., IDH vs. wild-type) and did not cross-verify potential biomarkers against these phenotypes (e.g., 1p19q codeletion or malignancy).

The main purpose of the initial analysis was to determine whether coupling the proposed sample-preparation method with high-performance analytical instrumentation would enable the differentiation of various histological types of brain tumors without requiring standard sample pretreatment protocols (i.e., weighting and homogenizing), which are typically performed in the laboratory, thus precluding on-site sampling, sample preparation, and extraction. The main hypothesis of the current study is that SPME is a simple sampling and extraction method that can be used for on-site brain tumor analysis. If this hypothesis is confirmed, future research can test the viability of this approach as an intraoperative tool for the diagnostic analysis of human brain tumors when coupled to on-site instrumentation. For now, however, the SPME fibers were used to sample the tumors in the hospital just after resection, with instrumental analysis being completed in a laboratory at a later date.

The present study aimed to determine whether metabolic phenotyping—and thus the analysis of biomarkers in the future—can be performed on fresh, intact tumor tissue using a simple on-site extraction method, and how the resultant data correlates with current reports on gliomas. Therefore, before beginning an in-depth investigation of glioma phenotypes, preliminary experiments were conducted to compare the metabolomes of gliomas and meningiomas, which are brain tumors of different origin, in order to verify the proposed sampling method’s suitability for the designed studies. The results of the supervised and unsupervised models showed the proposed sampling method achieved satisfactory separation of the studied groups and good validation, thus providing a proof-of-concept that optimized SPME-LC-HRMS protocols can be applied for metabolomic studies of brain tumors (Figure 3, Table S7). Although the identification of biomarkers differentiating meningiomas and gliomas is not meaningful from a diagnostics point of view, the discriminant metabolites identified via multi- and univariate analyses were nevertheless compared. As the two statistical approaches gave different results, both methods were used for further analysis. The findings of the univariate analyses indicated that aspartic acid and lysine are more down-regulated in gliomas compared to meningiomas, while the LASSO evaluations revealed that several molecules are up-regulated in gliomas, including glycerylphosphorylcholine (*p* < 0.0005), creatine (*p* < 0.0005), and citruline (*p* < 0.005) (Table S9). Notably, an ANOVA found that cystathionine had the highest [HGG+LGG/MEN] ratio (156.75) (Table S14).

With the preliminary test completed, an analysis focusing exclusively on gliomas was conducted. In this study, the obtained samples were profiled with respect to their genetic and histological features, as this approach demonstrated that some metabolites could appear in profiles reflecting two different features, e.g., a given mutation and malignancy grade. This was the case for both aminolevulinic acid (Tables S10 and S11) and amionoadipic acid (Tables S12 and S13). This approach was employed for both statistical methods used in this analysis, namely multivariate analysis (i.e., PCA and LASSO-OPLS-DA) and univariate analysis (i.e., ANOVA). Notably, the metabolites selected via LASSO to construct the OPLS-DA model differed from those deemed to have statistical significance in the ANOVA analysis. Contrary to ANOVA, the metabolites in the chemometric models may not be significant individually, but their corporate constellation nevertheless enables a given sample to be assigned to the appropriate group. In their review, Saccenti et al. compared the usefulness of uni- and multivariate analyses in metabolomics studies and discussed the advantages and disadvantages of each approach [43]. Based on the examples they examined, they concluded that the two types of methods are useful, as they provide various types of information and complementary biomarkers; therefore, the coupling of these strategies was highly advised. While the use of single biomarkers is more common in medical diagnostics, there are other analytical strategies that provide rapid sample identification based on molecular (spectral) matching with stored databases and built models (i.e., iKnife) [15]. This proves that the identification of individual compounds may not be critical, and that samples can be successfully assigned based on a comparison of their entire profiles with a given built model. The above-noted univariate and multivariate approaches were also employed to identify compounds of statistical significance with respect to the differentiation of the metabolic profiles of high- and low-grade gliomas, gliomas with and without IDH mutations, and gliomas with and without 1p19q codeletion. Moreover, pathway analysis was also conducted to identify metabolites related to metabolic networks, as this can provide greater insight into changes that occur in the underlying biochemistry of patients with gliomas. The pathway analysis was based on significantly important metabolic features from all analyzed cohorts of patients with brain tumors, which were determined using data from a PFP column in positive ionization mode (Figure 6C and Figure 8C). The results revealed that carnitine and L-2-aminoadipic acid were the compounds most associated with lysine degradation, while creatine and proline were the two metabolites most associated with arginine and proline metabolism (Figure 6C). The most important metabolic pathways indicating the presence of 1p19q codeletion were nicotinate and nicotinamide metabolism (associated with nicotinamide) and cysteine and methionine metabolism (related with cystathionine) (Figure 8C).

The findings of an ANOVA test also revealed that cystathionine, which was previously mentioned as a discriminant for gliomas and meningiomas, was also up-regulated in patients with codeletion of 1p19q (*p* < 0.05; [no-deletion/deletion] ratio: 0.22) (Table S15). In addition, a comparison of patients with high-grade and low-grade gliomas via LASSO (Table S10) and ANOVA (Table S12) identified 2-aminoadipic acid (2-AAD) (*p* < 0.05; [HGG/LGG] ratio 15.89) as a highly significant metabolite differentiating the two groups (*p* < 0.05; [HGG/LGG] ratio 11.33). Furthermore, up-regulated 2-AAD was also present in patients without IDH mutation (*p* < 0.005; [no-IDH/IDH] ratio 16.97). Although 2-AAD is present in the human brain [44] and cerebrospinal fluid (CSF) at low levels [45,46], findings have shown that it is present at significantly higher levels in glioma patients [41]. Furthermore, 2-AAD has been proven to be a glio- and neurotoxin, with some studies showing that it manifests its preferential toxicity towards astrocytes [47]. Notably, 2-AAD has been identified as a potential biomarker of cancer [46], and it has recently been detected by mass spectrometry in glioma tissues following surgery. These findings revealed an inverse relationship between patient survival and the signal intensity of 2-AAD in glioblastoma multiforme cells with stem-like properties, suggesting that 2-AAD may be able to play a key role in the prognosis of GBM tumors [47].

Similarly, propionylcarnitine levels were found to be highly significant in differentiating between patients with high- and low-grade grade gliomas (*p* < 0.001; [HGG/LGG] ratio: 14, 36) (Table S12) as well as between those with and without IDH mutations (*p* < 0.01; [no-IDH/IDH] ratio 11.05) (Table S13). These results are in agreement with previous reports showing that patients with the IDH 1 mutation are less able to produce α-ketoglutarate (α-KG, oxoglutaric acid) and NADPH, thus inhibiting fatty acid oxidation [48]. Mutations in IDH1 and IDH2 result in the conversion of 2-oxoglutarate to D-2-hydroxyglutarate. Consequently, high levels of D-2-hydroxyglutarate (D-2-HG) inhibit γ-butyrobetaine 1 hydroxylase, which is the last enzymatic step in carnitine biosynthesis [49]. Carnitine facilitates the transportation of fatty acids into the mitochondria, where its acyl derivatives are formed via an esterification process during the β-oxidation of fatty acids. The amount of carnitine derivatives, including propionylcarnitine, is lower in IDH1 and IDH2 mutants [50,51], which can contribute to increased fatty acid synthesis, decreased oxidation of certain fatty acids, and accelerated proliferation of cancer cells and tumor growth. Significantly, the proposed protocol was unable to detect α-ketoglutarate, 2-oxoglutarate, and D-2-hydroxyglutarate, which indicates the need for further optimization to ensure that it is able to detect these and other undetected diagnostically important compounds in future analyses. Nonetheless, the proposed protocol was able to identify the alanine, aspartate, glutamine pathway, which includes 2-oxoglutarate, as one of the most important metabolic pathways influencing the occurrence of IDH mutations. Therefore, while the proposed method did not directly indicate the presence of 2-oxoglutarate, it was able to identify gamma-aminobutyric acid, which is one of the elements in the above-described pathway (Figure 6C). Moreover, recent work in which SPME-LC-MS was applied to profile carnitine and 22 acylcarnitines in gliomas as a part of global lipidomic phenotyping found characteristic relations between short- and long-chain carnitine esters and tumor malignancy and mutations [52]. This finding also indicates that the application of various SPME protocols can enable the acquisition of a wide range of metabolites with different physical-chemical properties, and that the use of this method for future targeted analyses simply relies on the proper optimization of the coating chemistry and desorption mixture.

As mentioned above, IDH mutations, which are specific to astrocytomas and oligodendrogliomas, are characterized by the accumulation of 2-hydroxyglutarate in tumor cells [53,54], while 1p/19q codeletion is observed only on the oligodendroglial histologic subtype and is highly associated with cystathionine levels due to the higher expression of cystathionine-b-synthase (CBS) [55]. It has been proven that higher expression of CBS is associated with better prognoses in IDH-mutated 1p/19q-codeleted gliomas [56]. In the present study, the univariate analysis indicated that the concentration of cystathionine was almost five times higher in codeleted gliomas compared to the wild-type. This is consistent with Branzoli et al.’s findings, which showed the selective accumulation of cystathionine in codeleted gliomas in brain tissue samples in vivo, and selective vulnerability of codeleted gliomas to serine and glutathione depletion [57].

Global metabolomics provides a unique opportunity to characterize the entire phenotype of a given tissue. However, most analyses of gliomas focus on a specific variable as a diagnostic reference (i.e., histological type or the presence of IDH mutation) or on identifying potential biomarker(s). As a result, these studies do not account for other variables that are components of the sample’s phenotype. In the current study, the genetic tests conducted in the hospital laboratory were performed in sets targeting a few different mutations, independently of the tumor’s histological type. For instance, codeletion 1p19q, which is characteristic of oligodendrogliomas, was also determined in samples collected from patients with astrocytomas. The results showed that certain features, presented as significant metabolites, are dominant in the characteristics of gliomas and appear as differentiators of both IDH/no-IDH, deletion/no-deletion, and low-/high-grade-malignancy (e.g., threonine) mutants. This result is due to the coexistence of various analyzed features of gliomas in the same sample (i.e., different mutations and specific histological type). Taking the above into account—as well as the fact that, in current clinical practice, therapeutic strategy is based on the analysis of all accessible biomarkers (i.e., histological and genetic)—the best approach for future metabolomics research aiming to identify molecular diagnostic biomarkers of gliomas would be to characterize phenotypes containing information about all clinically important features, rather than focusing on biomarkers corresponding to individual mutations. The characterization of these phenotypes could prove invaluable to doctors, as it could allow them to develop and propose more personalized treatment strategy for patients. However, the cohorts used in such studies must be sufficiently large to provide statistically significant and reliable results.

The main limitation of this study was the small cohort that was used. Despite this limitation, we were able to verify SPME’s potential as a simple on-site sampling and extraction method for use in metabolomics workflows, which was the primary goal of this work. The short sampling/extraction times enabled by SPME eliminate sample preparation as a bottleneck in tumor tissue analytical protocols aimed at the extraction of a representative set of metabolites instead of the widest possible coverage of analytes. Another significant limitation of this work was the inability to identify some compounds considered essential from the point of view of glioma biochemistry point (e.g., 2-KG) or neoplasms in general (e.g., NADPH/NADH, GSH/GSSG). As mentioned before, this limitation should be addressed in future work by optimizing the protocol for target compounds.

The studies presented herein aimed to prove the applicability of SPME to profile brain tumors with respect to its diagnostic potential. However, the overall analysis time for the presented untargeted analysis does not meet expectations for intra-surgical protocol. Therefore, it needs to be emphasized that there are ongoing parallel studies complementing the investigations on device/protocol modifications and instrumental analysis following on-site sampling to provide the medical practitioners with ready-to-use solution for rapid determination of target biomarkers. In recent work, a device which on one hand enables to shorten the extraction process to 4 min and to perform extraction from two brain locations simultaneously on the other was presented [58]. This strategy is intended to be used eventually for the analysis of brain tumors and healthy brain tissue in the given patient at the same time. Moreover, an approach using regular SPME probe without implementing any modification to the device itself was used for the assessment of kidney graft quality during transplantation with only 10 min extraction time [59] and for the determination of the concentration of doxorubicin in pig and human lungs in vivo along with untargeted metabolomic profiling of the lung tissue with a 20 min extraction time [60]. The examples above show that modifications to the protocol related to the time of extraction are possible and have already been tested on different applications. With regard to instrumental analysis, there are many options for the direct coupling of the SPME probe with analytical instrumentation, particularly mass spectrometers [61]. A strategy which seems to be specifically suitable for this purpose is the microfluidic open interface, presented lately for the fast monitoring of drugs [62,63,64].

## 5. Conclusions

This study provides evidence supporting solid-phase microextraction’s viability as a fast and simple tool for extracting representative metabolites from intact tumor tissue directly in the operating room. However, this method could be improved for future applications by modifying the protocol to complement the range of detected metabolites with other compounds of diagnostic relevance. A comparison of the obtained metabolic phenotyping with routinely used tests indicated that a large-scale study might enable the description of phenotypes containing information about genetic and histological factors in addition to other features of diagnostic value.

## Figures and Tables

**Table 1:** Characteristics of patients participated in the study.

Tumor Characteristics	Number of Patients
Total number of patients	38
Sex	
Male	15
Female	23
Tumor subtypes and grades (total number)	38
Meningiomas	18
Meningioma grade I	15
Atypical meningioma grade II	2
Anaplastic meningioma grade III	1
Diffuse astrocytic and oligodendroglial tumors	19
Diffuse astrocytoma, IDH mutant	7
Anaplastic astrocytoma, IDH mutant	2
Glioblastoma, IDH wildtype	9
Ependymal tumors	1
Anaplastic ependymoma	1
Other astrocytic tumors	1
Pilocytic Astrocytoma	1
Oligoastrocytoma 1p/19q-codeted	6
Glioblastoma 1p/19q-codeleted	1

## Data Availability

Not applicable.

## Supplementary Materials

The following are available online at https://www.mdpi.com/article/10.3390/cancers14020312/s1, Figure S1: PLS-DA scores plots presenting differences between groups of patients with and without IDH mutations. Patients with IDH mutations—blue circles; patients without IDH mutations—green squares. Analyses were performed on an HILIC column in negative ionization mode (Model 5), an HILIC column in positive ionization mode (Model 6), and a PFP column in negative ionization mode (Model 7). Figure S2: PLS-DA score plots showing differences between groups of patients with and without codeletion 1p19q. Patients with codeletion—blue circles; patients without codeletion—green squares. Analyses were performed on an HILIC column in negative ionization mode (Model 8); an HILIC column in positive ionization mode (Model 9); and a PFP column in negative ionization mode (Model 10). Table S1: Comparison of the number of features, annotated compounds, and identified metabolites in patients with brain tumors using PFP and HILIC columns in positive and negative ionization modes. Table S2: Compounds identified in patients with brain tumors using Chem Spider database. Data for PFP column, ESI+. Table S3: Statistical performance of PLS-DA model for meningioma and glioma. Data for PFP column in positive ionization mode. Table S4: Statistical performance of OPLS-DA model for patients with/without IDH, with/without codeletion 1p19q, and high- and low-grade gliomas. Data for PFP column in ESI+. OPLS-DA models. Table S5: Panel of compounds representing differences between gliomas and meningiomas selected by LASSO using a PFP column in positive ionization mode. Table S6: Compounds differentiating gliomas and meningiomas based on ANOVA using PFP column in positive ionization mode. Table S7: Panel of compounds representing differences between high-grade- (stage III and IV) and low-grade-malignancy gliomas (stage I and II) selected by LASSO using a PFP column in positive ionization mode. Table S8: Compounds differentiating high-grade- and low-grade-malignancy gliomas based on ANOVA using a PFP column in positive ionization mode. Table S9: Panel of compounds representing differences between patient with and without isocitrate dehydrogenase mutations selected by LASSO using a PFP column in positive ionization mode. Table S10: Comparison of masses selected by lasso in the analysis of patients with/without IDH using different columns and ionization modes. Table S11: Statistical performance of PLS-DA model for patients with and without IDH mutation. Data for PFP column acquired in ESI- mode; data for HILIC column acquired in ESI+ and ESI- modes. Table S12: Compounds differentiating patients with and without isocitrate dehydrogenase mutations based on ANOVA using a PFP column in positive ionization mode. Table S13: Panel of compounds representing differences between patients with and without 1p19q codeletion selected via the LASSO method using a PFP column in positive ionization mode. Table S14: Comparison of masses selected via LASSO for the analysis of patients with/without codeletion using different columns and ionization modes. Table S15: Statistical performance of PLS-DA for patients with and without 1p19q codeletion. Data for PFP column acquired in ESI- mode; data for HILIC column acquired in ESI+ and ESI- mode. Table S16: Compounds differentiating patients with and without 1p19q codeletion selected via ANOVA using a PFP column in positive ionization mode.
