# Supplementary material for: Metabolomic Phenotyping of Gliomas: What Can We Get with Simplified Protocol for Intact Tissue Analysis?
Source: Cancers (Basel). 2022 Jan 9;14(2):312. doi: 10.3390/cancers14020312 (PMC8773998; doi:10.3390/cancers14020312)
Supplement: Supplementary file 1 [file cancers-14-00312-s001.zip › cancers-1510543-supplementary.pdf]

Supplementary information Table of Contents

Table of Contents 1

Tables 3

Table S1. Comparison of the number of features and annotated compoundsbased on ChemSpider search in patients with brain tumors using PFP and HILIC columns in positive and negative ionization modes. 3

Table S2: Compounds identified in patients with brain tumors using Chem Spider database. Data for PFP column, ESI+.. 3

Table S3: Statistical performance of PLS-DA model for meningioma and glioma. Data for PFP column in positive ionization mode. 7

Table S4. Statistical performance of OPLS-DA model for patients with/without IDH, with/without codeletion 1p19q, and high- and low-grade gliomas. Data for PFP column in ESI+. OPLS-DA models. 8

Table S5. Panel of compounds representing differences between gliomas and meningiomas selected by LASSO using a PFP column in positive ionization mode 9

Table S6. Compounds differentiating gliomas and meningiomas based on ANOVA using PFP column in positive ionization mode. 10

Table S7. Panel of compounds representing differences between high-grade- (stage III and IV) and low-grade-malignancy gliomas (stage I and II) selected by LASSO using a PFP column in positive ionization mode. 12

Table S8. Compounds differentiating high-grade- and low-grade-malignancy gliomas based on ANOVA using a PFP column in positive ionization mode. .. 14

Table S9. Panel of compounds representing differences between patient with and without isocitrate dehydrogenase mutations selected by LASSO using a PFP column in positive ionization mode. 15

Table S10. Comparison of masses selected by LASSO in the analysis of patients with/without IDH using different columns and ionization modes. 16

Table S11. Statistical performance of PLS-DA model for patients with and without IDH mutation. Data for PFP column acquired in ESI- mode; data for HILIC column acquired in ESI+ and ESI- modes. 17

Table S12. Compounds differentiating patients with and without isocitrate dehydrogenase mutations based on ANOVA using a PFP column in positive ionization mode. 18

Table S13. Panel of compounds representing differences between patients with and without 1p19q codeletion selected via the LASSO method using a PFP column in positive ionization mode. 19

Table S14. Comparison of masses selected via LASSO for the analysis of patients with/without codeletion using different columns and ionization modes. 20

Table S15. Statistical performance of PLS-DA for patients with and without 1p19q codeletion. Data for PFP column acquired in ESI- mode; data for HILIC column acquired in ESI+ and ESI- mode. 21

Table S16. Compounds differentiating patients with and without 1p19q codeletion selected via ANOVA using a PFP column in positive ionization mode. . 22

Figures 23

Figure S1. PLS-DA scores plots presenting differences between groups of patients with and without IDH mutations. Patients with IDH mutations—blue circles; patients without IDH mutations—green squares. Analyses were performed on an HILIC column in negative ionization mode (Model 5), an HILIC column in positive ionization mode (Model 6), and a PFP column in negative ionization mode (Model 7). 23

Figure S2. PLS-DA score plots showing differences between groups of patients with and without codeletion 1p19q. Patients with codeletion—blue circles; patients without codeletion—green squares. Analyses were performed on an HILIC column in negative ionization mode (Model 8); an HILIC column in positive ionization mode (Model 9); and a PFP column in negative ionization mode (Model 10). 24

Table S1. Comparison of the number of features and annotated compounds based on ChemSpider searchin patients with brain tumors using PFP and HILIC columns in positive and negative ionization modes

	Ionization mode			
	PFP (+)	PFP (-)	HILIC (+)	HILIC (-)
Number of features	338	201	124	113
Number of annotated compounds based on ChemSpider search	200	44	51	31

Table S2. Compounds identified in patients with brain tumors using Chem Spider database. Data for PFP column, ESI+.

**Name**	**Formula**	**Molecular Weight**	**Retention Time [min]**
dimethylformamide	C3 H7 N O	73.05275	1.46
FLUORACETIC ACID	C2 H3 F O2	78.01193	1.449
Proline	C5 H9 N O2	83.03732	1.462
Phosphoric acid	H3 O4 P	97.9772	18.843
L-(-)-Serine	C3 H7 N O3	105.04292	1.356
Hypotaurine	C2 H7 N O2 S	109.01978	1.094
3,4-Diaminopyridine	C5 H7 N3	109.06431	2.045
Uracil	C4 H4 N2 O2	112.02752	1.459
Creatinine	C4 H7 N3 O	113.05925	3.768
Proline	C5 H9 N O2	115.06356	1.459
tuaminoheptane	C7 H17 N	115.13629	11.37
tuaminoheptane	C7 H17 N	115.13629	11.057
tuaminoheptane	C7 H17 N	115.13629	11.195
tuaminoheptane	C7 H17 N	115.13635	10.972
tuaminoheptane	C7 H17 N	115.13636	10.861
1-Ethyl-1-nitrosourea	C3 H7 N3 O2	117.05405	1.971
Indole	C8 H7 N	117.05802	22.355

L-(+)-Valine	C5 H11 N O2	117.0791	3.293
alpha-methylstyrene	C9 H10	118.0786	17.784
L-(-)-Threonine	C4 H9 N O3	119.05841	1.425
Phenylethanolamine	C8 H9 N	119.07373	9.566
Nicotinamide	C6 H6 N2 O	122.04822	4.052
Taurine	C2 H7 N O3 S	125.01471	1.079
L-Pyroglutamic acid	C5 H7 N O3	129.04252	1.381
L-Pyroglutamic acid	C5 H7 N O3	129.04261	1.467
DL-Lysine	C6 H14 N2 O2	129.07902	2.039
Aminolevulinic acid	C5 H9 N O3	131.05824	1.275
Creatine	C4 H9 N3 O2	131.06953	2.467
L-(+)-Leucine	C6 H13 N O2	131.0947	7.709
Leucine	C6 H13 N O2	131.0947	8.13
L-(-)-Asparagine	C4 H8 N2 O3	132.0535	1.334
L-Aspartic acid	C4 H7 N O4	133.03749	1.333
Cinnamyl alcohol	C9 H10 O	134.07322	17.783
Cinnamyl alcohol	C9 H10 O	134.07324	16.438
Adenine	C5 H5 N5	135.05461	7.785
Acetanilide	C8 H9 N O	135.06846	7.852
Hypoxanthin	C5 H4 N4 O	136.03852	1.461
Hypoxanthin	C5 H4 N4 O	136.03854	7.042
4-Nitroaniline	C6 H6 N2 O2	138.04296	7.644
DL-Lysine	C6 H14 N2 O2	146.10547	2.042
DL-Glutamic acid	C5 H9 N O4	147.05314	1.47
Triethanolamine	C6 H15 N O3	149.10526	2.582
Guanine	C5 H5 N5 O	151.04945	1.46
Paracetamol	C8 H9 N O2	151.06336	8.207
Xanthine	C5 H4 N4 O2	152.03336	1.459
(+/-)-Camphor	C10 H16 O	152.12021	16.701

L-Histidine	C6 H9 N3 O2	155.06954	2.044
L-(+)-Citrulline	C6 H13 N3 O3	158.06912	1.466
L-2-Aminoadipic acid	C6 H11 N O4	161.06877	1.722
DL-Carnitine	C7 H15 N O3	161.10523	4.298
L-Tyrosine	C9 H11 N O3	164.04742	7.849
L-Phenylalanine	C9 H11 N O2	165.07907	9.565
DL-Arginine	C6 H14 N4 O2	174.11169	2.368
L-(+)-Citrulline	C6 H13 N3 O3	175.09567	1.462
4-Methoxycinnamic acid	C10 H10 O3	178.06298	22.296
Hexitol	C6 H14 O6	182.07899	1.113
Indoleacrylic acid	C11 H9 N O2	187.06337	12.284
Tryptophanamide	C11 H13 N3 O	203.10593	12.09
Acetyl-L-carnitine	C9 H17 N O4	203.11586	9.595
Maraniol	C12 H12 O3	204.0787	20.501
4-(1,2-Dihydroxy-2-propanyl)-1-methyl-1,2-cyclohexanediol	C10 H20 O4	204.13606	13.966
4-(1,2-Dihydroxy-2-propanyl)-1-methyl-1,2-cyclohexanediol	C10 H20 O4	204.13606	14.007
4-(1,2-Dihydroxy-2-propanyl)-1-methyl-1,2-cyclohexanediol	C10 H20 O4	204.13618	14.069
(2Z)-2-Benzylidene-1-heptanol	C14 H20 O	204.15131	21.513
2-Decylfuran	C14 H24 O	208.18274	16.701
UNII:240I539PWQ	C5 H14 N O6 P	215.05573	1.075
1,5-Dimethyl-4-(methylamino)-2-phenyl-1,2-dihydro-3H-pyrazol-3-one	C12 H15 N3 O	217.12149	12.97
Propionylcarnitine	C10 H19 N O4	217.13134	11.713
2,2,4,4,6,6-Hexamethyl-1,3,5-trithiane	C9 H18 S3	222.05627	15.4
2,2,4,4,6,6-Hexamethyl-1,3,5-trithiane	C9 H18 S3	222.05634	24.971
Cystathionine	C7 H14 N2 O4 S	222.06734	1.487
N-(tert-Butoxycarbonyl)-L-leucine	C11 H21 N O4	231.14696	14.741
Acetyl-L-tryptophanamide	C13 H15 N3 O2	245.1164	8.289
Tolperisone	C16 H23 N O	245.17782	21.509
Pindolol	C14 H20 N2 O2	248.15229	20.736

7-Methyl-2,6,9,12-tetraoxahexadecane	C13 H28 O4	248.19857	14.435
Tolmetin	C15 H15 N O3	257.10253	1.199
UNII:Z5JO63XGNK	C17 H26 O2	262.19314	20.828
Tributyl phosphate	C12 H27 O4 P	266.16463	18.839
2-Amino-9-(2-deoxypentofuranosyl)-3,9-dihydro-6H-purin-6-one	C10 H13 N5 O4	267.0966	7.781
Arabinosylhypoxanthine	C10 H12 N4 O5	268.08055	7.042
Kaur-16-ene	C20 H32	272.24993	23.057
gamma-Glu-gln	C10 H17 N3 O6	275.11152	1.459
Dibutyl phthalate	C16 H22 O4	278.15166	20.504
(9E,12E,15E)-9,12,15-Octadecatrienoic acid	C18 H30 O2	278.22443	21.514
Linoleamide	C18 H33 N O	279.25604	20.415
Phaseic acid	C15 H20 O5	280.13094	17.787
Methyl 15-cyanopentadecanoate	C17 H31 N O2	281.23534	21.031
8-hydroxy-deoxyguanosine	C10 H13 N5 O5	283.09143	1.463
Stearamide	C18 H37 N O	283.28729	21.915
Stearamide	C18 H37 N O	283.2873	22.756
all-trans-retinal	C20 H28 O	284.21387	21.886
N-Tetradecanoylglycine	C16 H31 N O3	285.23027	20.265
N,N-Bis(2-hydroxyethyl)dodecanamide	C16 H33 N O3	287.24578	24.566
octyl methoxycinnamate	C18 H26 O3	290.18799	22.299
1-Hexadecanoylpyrrolidine	C20 H39 N O	309.30298	22.17
1-(14-methylhexadecanoyl)pyrrolidine	C21 H41 N O	323.31873	22.634
(2E,4E,12Z)-N-Isobutyl-2,4,12-octadecatrienamide	C22 H39 N O	333.30297	21.873
(2E,4Z)-N-Isobutyl-2,4-octadecadienamide	C22 H41 N O	335.31855	22.439
(2E,4Z)-N-Isobutyl-2,4-octadecadienamide	C22 H41 N O	335.31859	19.905
Docosanamide	C22 H45 N O	339.34988	23.9
Eplerenone	C24 H30 O6	414.20424	17.78
Cefazolin	C14 H14 N8 O4 S3	454.03011	10.379
1-stearoyl-2-arachidonoyl-sn-glycero-3-phosphoethanolamine zwitterion	C43 H78 N O8 P	767.54611	25.486

Table S3. Statistical performance of PLS-DA model for meningioma and glioma. Data for PFP column in positive ionization mode.

**Parameter**	**Model 4**	
**Classes**	Men	LGG+HGG
**Data preprocessing**	Log_{10}, autoscale	
**N**	17	19
**Number of LV**	2	
**RMSEC**	0.106	0.106
**RMSECV**	0.133	0.133
**R^2(calibration)**	0.954	0.954
**R^2(CV)**	0.928	0.928
**NMC**	0	
**AUROC**		
**Calibration**	1.000	1.000
**Cross-validation**	1.000	1.000

AUROC – Area Under the Operating Receiver; NMC – Number of misclassification; RMSEC – Root Mean Square Error of Calibration; RMSECV – Root Mean Square Error of Cross-validation;

Table S4. Statistical performance of OPLS-DA model for patients with/without IDH, with/without codeletion 1p19q, and high- and low-grade gliomas. Data for PFP column in ESI+. OPLS-DA models.

**Parameter**	**Model 1**		**Model 2**		**Model 3**	
**Classes**	LGG	HGG	IDH	lack of IDH	deletion	without deletion
**Data preprocessing**	Log_{10}, autoscale		Log_{10}, autoscale		Log_{10}, autoscale	
**N**	7	12	10	9	7	12
**Number of LV**	4		3		2	
**RMSEC**	0.125	0.125	0.096	0.096	0.651	0.401
**RMSECV**	0.491	0.491	0.203	0.203	0.679	0.462
**R^2(calibration)**	0.932	0.932	0.963	0.963	0.894	0.894
**R^2(CV)**	0.163	0.163	0.836	0.836	0.714	0.714
**NMC**			0		0	
**AUROC**<br>**Calibration**<br>**Cross-validation**	 1.000<br>0.738	 1.000<br>0.736	 1.000<br>1.000	 1.000<br>1.000	 1.000<br>1.000	 1.000<br>1.000

AUROC – Area Under the Operating Receiver; NMC – Number of misclassification; RMSEC – Root Mean Square Error of Calibration; RMSECV – Root Mean Square Error of Cross-validation;

Table S5. Panel of compounds representing differences between gliomas and meningiomas selected by LASSO using a PFP column in positive ionization mode.

Molecular weight	p-value	p-value correction	Ratio [HGG+LGG/Men]	Identification
89.0481	<0.00001	<0.0005	0.25	-
132.0535	<0.00005	<0.0005	0.25	Ureidopropionic acid[#]<br>Asparagine[#]<br>Glycyl-glycine[#]<br>N-Carbamoylsarcosine[#]
257.1025	<0.00005	<0.0005	22.13	Glycerylphosphorylcholine[#]
152.0333	<0.0005	<0.001	3.91	**Xanthine**[#]<br>Oxypurinol[#]<br>6,8-Dihydroxypurine[#]
146.1055	<0.0005	<0.001	0.37	Lysine[*]
131.0694	<0.0005	<0.001	3.06	Creatine[*]
289.127	<0.0005	<0.005	0.08	ophthalmic acid[#]
133.0375	<0.001	<0.005	0.22	Aspartic acid[*]
175.0956	<0.005	<0.005	1.66	Citruline[*]
343.9939	<0.005	<0.005	0.42	-
232.9746	<0.005	<0.01	0.26	-
125.0147	<0.005	<0.01	0.39	Taurine[#]
280.1419	<0.01	<0.01	2.79	**Valyltyrosine**[#]<br>**Tyrosyl-Valine**[#]
384.1217	<0.01	<0.01	2.08	**S-Adenosylhomocysteine** [#]
245.1622	<0.01	<0.05	10.49	2-Methylbutyroylcarnitine[#]<br>Isovalerylcarnitine[#]<br>Pivaloylcarnitine[#]
245.1161	<0.05	<0.05	0.09	
262.0738	<0.1	<0.1	0.28	-

note: [*]compounds identified based on fragmentation spectra; [#]compounds identified based on accurate parent mass

Table S6. Compounds differentiating gliomas and meningiomas based on ANOVA using PFP column in positive ionization mode.

Molecular weight	p-value	p-value correction	Ratio [HGG+LGG/Men]	Identification
222.0673	<0.01	>0.5	156.75	Cystathionine*
133.0375	<0.05	>0.5	0.22	Aspartic acid*
174.1116	<0.05	>0.5	0.26	Arginine*
146.1055	<0.05	>0.5	0.28	Lysine*
350.0957	<0.000000000001	<0.0000000005	0.003	
175.0480	<0.00000000005	<0.00000005	0.02	N-Acetyl-L-aspartic acid#
257.1025	<0.000000001	<0.0000005	0.04	L-alpha-Glycerylphosphorylcholine#
157.0374	<0.000000005	<0.0000005	0.06	Furaspor#
222.0672	<0.00000001	<0.000001	0.02	cystathionine#
197.0799	<0.00000005	<0.000005	0.05	N-Acetyl-L-histidine#
351.1098	<0.0000005	<0.00005	0.04	
245.1624	<0.000005	<0.0005	0.04	2-methylbutyrylcarnitine#
151.0494	<0.00001	<0.0005	28.29	Guanine#
289.1270	<0.00001	<0.0005	13.38	ophthalmic acid#
791.5458	<0.00001	<0.0005	0.14	
245.1622	<0.00001	<0.0005	0.07	2-methylbutyrylcarnitine#
231.1467	<0.00005	<0.0005	0.11	N-(tert-Butoxycarbonyl)-L-leucine#
89.0481	<0.00005	<0.001	4.21	
152.0333	<0.00005	<0.001	0.28	Xanthine#
89.0481	<0.0001	<0.005	3.72	
132.0535	<0.0005	<0.005	3.60	L-(-)-Asparagine#
304.0902	<0.0005	<0.005	0.17	Spaglumic acid#
133.0375	<0.0005	<0.01	4.00	L-(+)-Aspartic acid#
131.0694	<0.0005	<0.01	0.26	Creatine#
343.9939	<0.0005	<0.05	2.31	
103.0636	<0.0005	<0.05	0.13	gamma-Aminobutyric acid#

161.0687	<0.001	<0.02151498622798	7.32	.alpha.-Aminoadipic acid#
203.0794	<0.001	<0.05	2.26	N-acetyl-L-2-aminoadipic acid#
158.0690	<0.005	<0.05	0.51	oxiracetam#
113.0591	<0.005	<0.05	0.56	Creatinine#
146.1055	<0.005	<0.05	2.19	DL-Lysine#
129.0789	<0.005	<0.05	2.13	Vigabatrin#
663.1080	<0.005	<0.05	8.40	
203.1057	<0.005	<0.05	13.45	Tryptophanamide#
125.0147	<0.005	<0.05	2.65	Taurine#
226.0038	<0.005	<0.05	0.26	
174.1116	<0.005	<0.05	1.78	DL-Arginine#
149.0510	<0.005	<0.05	1.88	L-(-)-methionine#
490.1478	<0.005	<0.05	0.22	
175.0956	<0.005	<0.05	0.50	DL-Citrulline#
249.1111	<0.005	<0.05	1.26	Oxibendazole#
115.0635	<0.005	<0.05	2.55	L-Proline#
350.0957	<0.000000000001	<0.0000000005	0.003	

note: [*]compounds identified based on fragmentation spectra; [#]compounds identified based on accurate parent mass

Table S7. Panel of compounds representing differences between high-grade- (stage III and IV) and low-grade-malignancy gliomas (stage I and II) selected by LASSO using a PFP column in positive ionization mode.

Molecular weight	P-value	P-value correction	Ratio [HGG/LGG]	Identification
113.0591	<0.1	<0.5	0.51	Creatinine[*]
119.0583	<0.05	<0.5	2.01	Threonine[*]
131.0583	<0.05	<0.1	3.56	Aminolevulinic acid[*]
161.0687	<0.05	<0.5	15.89	2-aminoadipic acid[*]
203.0794	>0.5	>0.5	1.14	N-acetyl-L-2-aminoadipate(2-)[#]
225.1210	<0.01	<0.5	3.40	
242.9827	>0.5	>0.5	0.26	-
245.1622	<0.05	<0.5	8.21	2-Methylbutyroylcarnitine[#]<br>Isovalerylcarnitine[#]<br>Pivaloylcarnitine[#]
280.1420	<0.5	<0.5	1.61	Valyltyrosine[#]<br>**Tyrosyl-Valine**[#]<br>**Feruloyl-2-hydroxyputrescine**[#]
347.0628	<0.5	<0.5	1.29	Adenosine monophosphate[#]<br>2'-Deoxyguanosine 5'-monophosphate[#]<br>3'-AMP[#]<br>Adenosine 2'-phosphate[#]<br>2-hydroxy-dAMP[#]
377.8930	>0.5	>0.5	0.99	-
378.2299	<0.5	>0.5	0.72	Doxapram[#]
426.0875	<0.5	>0.5	1.26	Cysteineglutathione disulfide (Cys-SG) [#]
430.3079	>0.5	>0.5	5.77	**25-Hydroxyvitamin D3-26,23-lactol**[#]<br>**24-Oxo-1alpha,25-dihydroxyvitamin D3**[#]<br>**(25R)-3beta-hydroxycholest-5-en-7-one-26-oate**[#]

431.2880	<0.1	<0.5	4.60	-
684.1794	<0.5	>0.5	0.92	-

note: [*]compounds identified based on fragmentation spectra; [#]compounds identified based on accurate parent mass

Table S8. Compounds differentiating high-grade- and low-grade-malignancy gliomas based on ANOVA using a PFP column in positive ionization mode.

**Molecular weight**	**P-value**	**P-value correction**	**Ratio [HGG/LGG]**	**Identification**
165.0789	<0.05	>0.5	2.22	Phenylalanine*
115.0635	<0.005	<0.5	3.01	Proline*
161.0687	<0.05	>0.5	11.33	2-Aminoadipic acid*
217.1312	<0.001	<0.1	14.36	Propionylcarnitine*
181.0738	<0.05	>0.5	1.88	Tyrosine*
168.0282	<0.05	>0.5	3.08	Uric acid*
217.1312	<0.00001	<0.005	15	Propionylcarnitine#
161.1051	<0.0001	<0.01	7.3	Carnitine#
245.1624	<0.0001	<0.01	25.07	
117.0539	<0.00005	<0.01	17.06	1-ethyl-1-nitrosourea#
109.0200	<0.0005	<0.05	11.85	Hypotaurine#
115.0635	<0.0005	<0.05	3.27	Proline#
245.1623	<0.0005	<0.05	7.25	

note: [*]compounds identified based on fragmentation spectra; [#]compounds identified based on accurate parent mass

Table S9. Panel of compounds representing differences between patient with and without isocitrate dehydrogenase mutations selected by LASSO using a PFP column in positive ionization mode.

**Molecular weight**	**P-value**	**P-value correction**	**Ratio [IDH/no-IDH]**	**Identification**
89.0481	<0.5	<0.5	0.32	-
103.0999	<0.05	<0.05	0.26	Neurine[#]
113.0591	<0.05	<0.05	0.42	Creatinine[*]
119.0583	<0.05	<0.05	0.28	Threonine[*]
131.0583	<0.05	<0.05	7.89	Aminolevulinic acid[*]
161.1051	<0.01	<0.1	0.45	Carnitine[*]
225.1210	<0.01	<0.05	2.18	-
242.9827	<0.05	<0.05	1.51	-
245.1622	<0.05	<0.05	0.19	-
296.2825	<0.01	<0.05	0.48	-
426.0875	<0.05	<0.05	0.04	-
431.2880	<0.05	<0.05	0.48	-

note: [*]compounds identified based on fragmentation spectra; [#]compounds identified based on accurate parent mass

Table S10: Comparison of masses selected by LASSO in the analysis of patients with/without IDH using different columns and ionization modes.

IDH			
**HILIC_neg**	**HILIC_pos**	**PFP_neg**	**PFP_pos**
(*HILIC_neg_H38*)	(*HILIC_pos_H30*)	(*PFP_neg_H30*)	(*PFP_pos_D30*)
125.9971<br>136.0356<br>148.0358<br>157.0361<br>161.0676<br>182.0782<br>248.0481<br>256.2399<br>289.0015<br>344.0332	105.0792<br>113.0591<br>115.0635<br>129.1515<br>152.1313<br>165.0789<br>203.1157<br>215.0557<br>252.1472<br>255.2558<br>270.0509<br>287.1914<br>353.3039<br>925.2621<br>931.7542	112.0257<br>132.0522<br>136.0358<br>168.0272<br>196.0575<br>247.9576<br>255.1318<br>265.9417<br>272.902<br>328.2401<br>426.0884	89.0481<br>103.0999<br>113.0591<br>119.0583<br>131.0583<br>161.1051<br>225.121<br>242.9827<br>245.1622<br>296.2825<br>426.0875<br>431.288

Table S11. Statistical performance of PLS-DA model for patients with and without IDH mutation. Data for PFP column acquired in ESI- mode; data for HILIC column acquired in ESI+ and ESI- modes.

**Parameter**	**Model 5** (HILIC_neg)		**Model 6** (HILIC_pos)		**Model 7** (PFP_neg)	
**Classes**	IDH	lack of IDH	IDH	lack of IDH	IDH	lack of IDH
**N**	10	9	10	9	10	9
**Data preprocessing**	autoscale		autoscale		autoscale	
**Number of LV**	2		2		2	
**RMSEC**	0.198	0.198	0.205	0.205	0.128	0.128
**RMSECV**	0.321	0.321	0.374	0.374	0.231	0.231
**R^2(calibration)**	0.843	0.843	0.832	0.832	0.934	0.934
**R^2(CV)**	0.698	0.698	0.468	0.468	0.792	0.792
**NMC**						
**Calibration**	0	0	0	0	0	0
**Cross-validation**	1	0	2	2	0	0
**AUROC**						
**Calibration**	1.000	1.000	1.000	1.000	1.000	1.000
**Cross-validation**	0.856	0.856	0.922	0.922	1.000	1.000
**Permutation test (N = 150)**						
**Self-Prediction**	Passed		Failed		Passed	
**Cross-validated**	Failed		Failed		Passed	

Table S12. Compounds differentiating patients with and without isocitrate dehydrogenase mutations based on ANOVA using a PFP column in positive ionization mode.

**Molecular weight**	**P-value**	**P-value correction**	**Ratio [no-IDH /IDH]**	**Identification**
203.1156	<0.05	<0.5	3.22	Acetyl-L-carnitine*
165.0789	<0.01	<0.5	2.11	Phenylalanine*
168.0282	<0.01	<0.5	2.83	Uric acid*
115.0635	<0.01	<0.5	2.98	Proline*
161.0687	<0.005	<0.5	16.97	2-Aminoadipic acid*
217.1312	<0.01	<0.5	11.05	Propionylcarnitine*
242.9827	<0.00005	<0.0	0.08	
103.0636	<0.0005	<0.05	4.82	gamma-Aminobutyric acid#
109.0199	<0.0005	<0.05	35.06	
161.1051	<0.001	<0.05	6.31	Carnitine#
89.0481	<0.001	<0.05	3.3	

note: [*]compounds identified based on fragmentation spectra; [#]compounds identified based on accurate parent mass

Table S13. Panel of compounds representing differences between patients with and without 1p19q codeletion selected via the LASSO method using a PFP column in positive ionization mode.

**Molecular weight**	**P-value**	**P-value correction**	**Ratio [Deletion/no-deletion]**	**Identification**
89.0481	<0.001	<0.01	0.2	
103.0999	<0.0005	<0.01	0.43	Neurine[#]
119.0583	<0.005	<0.01	0.31	Threonine[*]
122.0481	<0.005	<0.01	0.37	**Nicotinamide (**Niacinamide)[#]
163.1207	<0.05	<0.1	2.42	
215.0557	<0.05	<0.1	4.46	sn-glycero-3-Phosphoethanolamine[#]<br>Glycerylphosphorylethanolamine [#]
244.0692	<0.5	<0.5	1.27	
248.1521	<0.5	<0.5	0.61	
332.292	<0.01	<0.05	1.9	
386.1724	<0.5	<0.5	1.69	
430.3079	<0.5	<0.5	0.13	25-Hydroxyvitamin D3-26,23-lactol[#]<br>24-Oxo-1alpha,25-dihydroxyvitamin D3[#]
612.1513	<0.05	<0.05	0.16	
612.1513	<0.5	<0.05	0.16	Oxidized glutathione (GSSG) [#]

note: [*]compounds identified based on fragmentation spectra; [#]compounds identified based on accurate parent mass

Table S14: Comparison of masses selected via LASSO for the analysis of patients with/without codeletion using different columns and ionization modes.

Deletion			
**HILIC_neg**	**HILIC_pos**	**PFP_neg**	**PFP_pos**
(*HILIC_neg_D31*)	(*HILIC_pos_D36*)	(*PFP_neg_D27*)	(*PFP_pos_D30*)
106.0248			
115.0258			89.0481
131.0931	105.0793		103.0999
134.9976	115.0634	134.9976	119.0583
136.0356	152.1313	196.0575	122.0481
148.0358	215.0557	222.067	163.1207
161.0676	243.2197	236.9487	215.0557
203.9937	248.1522	251.1519	244.0692
256.2399	255.2558	255.1318	248.1521
262.2295	267.0965	289.1275	332.2920
317.1239	287.1914	331.0766	386.1724
329.0526	315.2228	388.1221	430.3079
370.163	353.3039	612.1526	612.1513
484.1286	925.2621		684.1794

Table S15. Statistical performance of PLS-DA for patients with and without 1p19q codeletion. Data for PFP column acquired in ESI- mode; data for HILIC column acquired in ESI+ and ESI- mode.

**Parameter**	**Model 8** (HILIC_neg)		**Model 9** (HILIC_pos)		**Model 10** (PFP_neg)	
**Classes**	deletion	without deletion	deletion	without deletion	Deletion	without deletion
**N**	7	12	7	12	7	12
**Data preprocessing**	autoscale		autoscale		autoscale	
**Number of LV**	3		2		2	
**RMSEC**	0.153	0.153	0.148	0.148	0.102	0.102
**RMSECV**	0.805	0.805	0.319	0.319	0.192	0.192
**R^2(calibration)**	0.898	0.898	0.905	0.905	0.955	0.955
**R^2(CV)**	0.302	0.302	0.638	0.638	0.854	0.854
**NMC**						
**Calibration**	0	0	0	0	0	0
**Cross-validation**	2	1	1	0	0	0
**AUROC**						
**Calibration**	1.000	1.000	1.000	1.000	1.000	1.000
**Cross-validation**	0.905	0.905	1.000	1.000	1.000	1.000
**Permutation test (N = 150)**						
**Self-Prediction**	Failed		Passed		Passed	
**Cross-validated**	Failed		Passed		Passed	

Table S16. Compounds differentiating patients with and without 1p19q codeletion selected via ANOVA using a PFP column in positive ionization mode.

**Molecular weight**	**P-value**	**P-value correction**	**Ratio [no-deletion/deletion]**	**Identification**
222.0669	<0.05	<0.5	0.22	Cystathionine[*]
155.0683	<0.00000005	<0.000005	768.24	
612.1526	<0.00005	<0.0005	62.75	
268.0807	<0.00005	<0.0005	76.21	Arabinosylhypoxanthine[#]
354.1680	<0.0005	<0.005	0.01	Iclaprim[#]
417.1638	<0.001	<0.01	0.03	
272.9020	<0.005	<0.05	0.05	
112.0257	<0.005	<0.05	2.62	

note: [*]compounds identified based on fragmentation spectra; [#]compounds identified based on accurate parent mass

Figure S1. PLS-DA scores plots presenting differences between groups of patients with and without IDH mutations. Patients with IDH mutations—blue circles; patients without IDH mutations—green squares. Analyses were performed on an HILIC column in negative ionization mode (Model 5), an HILIC column in positive ionization mode (Model 6), and a PFP column in negative ionization mode (Model 7).

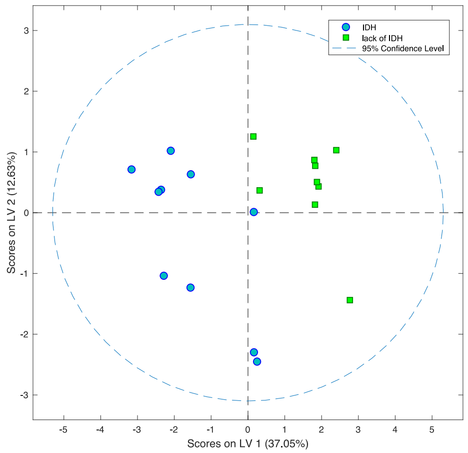

Model 5

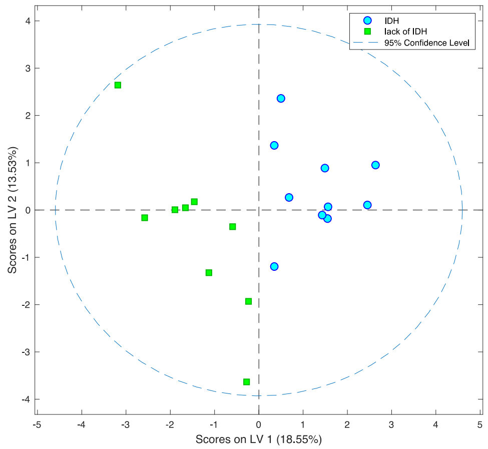

Model 6

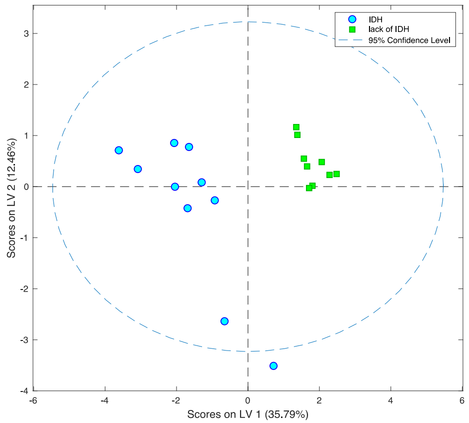

Model 7

Figure S2. PLS-DA score plots showing differences between groups of patients with and without codeletion 1p19q. Patients with codeletion—blue circles; patients without codeletion—green squares. Analyses were performed on an HILIC column in negative ionization mode (Model 8); an HILIC column in positive ionization mode (Model 9); and a PFP column in negative ionization mode (Model 10).

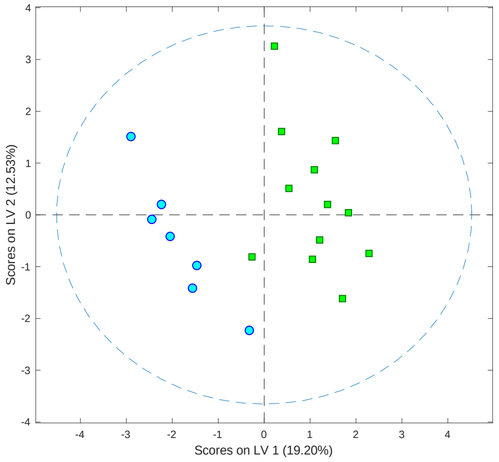

Model 8

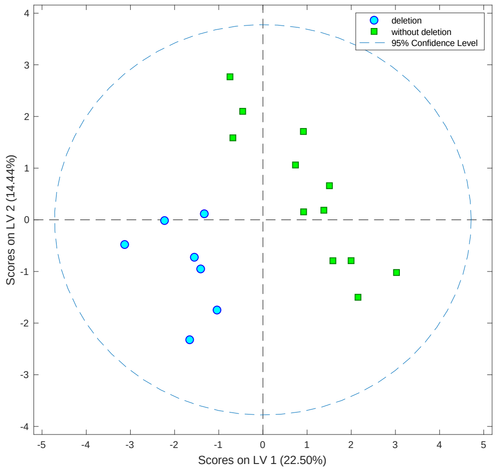

Model 9

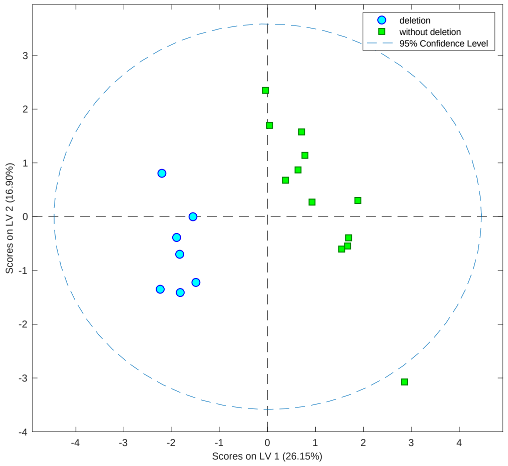

Model 10
